# Reliability of rapid diagnostic tests in diagnosing pregnancy-associated malaria in north-eastern Tanzania

**DOI:** 10.1186/1475-2875-11-211

**Published:** 2012-06-21

**Authors:** Daniel TR Minja, Christentze Schmiegelow, Mayke Oesterholt, Pamela A Magistrado, Stéphanie Boström, Davis John, Caroline Pehrson, Daniel Andersen, Philippe Deloron, Ali Salanti, Martha Lemnge, Adrian JF Luty, Michael Alifrangis, Thor Theander, John PA Lusingu

**Affiliations:** 1 National Institute for Medical Research, Tanga Centre, Tanga, Tanzania; 2 Centre for Medical Parasitology, Department of International Health, Immunology and Microbiology, University of Copenhagen and Department of Infectious Diseases, Copenhagen University Hospital, Copenhagen, Denmark; 3 Department of Medical Microbiology, Radboud University Nijmegen Medical Centre, Nijmegen, the Netherlands; 4 Kilimanjaro Christian Medical Centre, Kilimanjaro, Tanzania; 5 Institut de Recherche Pour le Développement (IRD), UMR 216 Mère et enfant face aux infections tropicales, Paris, France; 6 Faculté de Pharmacie, Université Paris Descartes, Sorbonne Paris Cité, France; 7 Department of Immunology, Wenner-Gren Institute, Stockholm University, Stockholm, Sweden; 8 Present address: IRD, UMR 216 Mère et enfant face aux infections tropicales, Paris, France

**Keywords:** Rapid diagnostic tests (RDTs), Reliability, Sensitivity, *Plasmodium falciparum*, Pregnancy-Associated Malaria (PAM), Microscopy, Polymerase chain reaction (PCR), Sub-microscopic infections, Pregnancy outcomes, Tanzania

## Abstract

**Background:**

Accurate diagnosis and prompt treatment of pregnancy-associated malaria (PAM) are key aspects in averting adverse pregnancy outcomes. Microscopy is the gold standard in malaria diagnosis, but it has limited detection and availability. When used appropriately, rapid diagnostic tests (RDTs) could be an ideal diagnostic complement to microscopy, due to their ease of use and adequate sensitivity in detecting even sub-microscopic infections. Polymerase chain reaction (PCR) is even more sensitive, but it is mainly used for research purposes. The accuracy and reliability of RDTs in diagnosing PAM was evaluated using microscopy and PCR.

**Methods:**

A cohort of pregnant women in north-eastern Tanzania was followed throughout pregnancy for detection of plasmodial infection using venous and placental blood samples evaluated by histidine rich protein 2 (HRP-2) and parasite lactate dehydrogenase (pLDH) based RDTs (Parascreen™) or HRP-2 only (Paracheck Pf® and ParaHIT®f), microscopy and nested *Plasmodium* species diagnostic PCR.

**Results:**

From a cohort of 924 pregnant women who completed the follow up, complete RDT and microscopy data was available for 5,555 blood samples and of these 442 samples were analysed by PCR. Of the 5,555 blood samples, 49 ((proportion and 95% confidence interval) 0.9% [0.7 -1.1]) samples were positive by microscopy and 91 (1.6% [1.3-2.0]) by RDT. Forty-six (50.5% [40.5 - 60.6]) and 45 (49.5% [39.4 – 59.5]) of the RDT positive samples were positive and negative by microscopy, respectively, whereas nineteen (42.2% [29.0 - 56.7]) of the microscopy negative, but RDT positive, samples were positive by PCR. Three (0.05% [0.02 - 0.2]) samples were positive by microscopy but negative by RDT. 351 of the 5,461 samples negative by both RDT and microscopy were tested by PCR and found negative. There was no statistically significant difference between the performances of the different RDTs.

**Conclusions:**

Microscopy underestimated the real burden of malaria during pregnancy and RDTs performed better than microscopy in diagnosing PAM. In areas where intermittent preventive treatment during pregnancy may be abandoned due to low and decreasing malaria risk and instead replaced with active case management, screening with RDT is likely to identify most infections in pregnant women and out-performs microscopy as a diagnostic tool.

## Background

Accurate diagnosis and prompt treatment of pregnancy-associated malaria (PAM) is essential to avert adverse pregnancy outcomes [1]. Detection of sub-microscopic infections is crucial in order to not only effect prompt treatment of asymptomatic cases, but also to identify and clear potential reservoirs of transmission [2,3] and to reduce malaria related morbidity and mortality. Presumptive treatment of malaria based on clinical diagnosis is relatively cheap but it is unreliable due to overlapping symptoms with non-malarial infections caused by viruses or bacteria [4] and could lead to over-diagnosis [5] as well. Wrong diagnoses may lead to presumptive medication and hence many patients may leave the health facility without the right treatment. Rational prescription of anti-malarials is not only important in saving on the cost of expensive drugs but it also prevents drug overuse that might result in the development of resistance [6]. Sub-microscopic infections during pregnancy might be associated with increased risk of adverse pregnancy outcomes including low birth weight babies and maternal anaemia [7,8]. Therefore, treatment of these infections may prevent potential risks of adverse pregnancy outcome [9].

PAM in the sub-Saharan Africa is caused by *Plasmodium falciparum*, and it is precipitated because VAR2CSA, member of the *P. falciparum* erythrocyte membrane protein 1 (*Pf*EMP-1) family expressed on the surface of infected erythrocytes (IEs) mediates sequestration of IEs in the intervillous spaces of the placenta by binding to chondroitin sulphate A (CSA) receptors [10,11]. The pathogenesis of PAM and its association with adverse pregnancy outcome [12,13], such as intrauterine growth retardation and low birth weight is not well understood but it is thought to be caused by impairment in nutrient transport to the foetus [14], with possible effects on growth regulating hormones [15] and trophoblast invasion [16]. As a result of placental sequestration it is often difficult to detect IEs in the peripheral blood using microscopy [17]. Furthermore, malarial infections are usually asymptomatic among adults in malaria endemic regions, decreasing the chances of clinical detection by using clinical algorithms. Primi- and secundigravidae as opposed to multigravidae are most affected as they lack sufficient previous exposure to allow the development of protective immunity [18,19].

Microscopic examination of blood smears has been the gold standard for malaria diagnosis but it is compromised by poor infrastructure and the need for individuals with expertise in microscopy who are not necessarily available in many health facilities in malaria endemic regions [20]. Furthermore, microscopy is not sensitive enough [5,9,21-24], it requires good quality reagents, well maintained microscopes, and is time consuming [25]. Studies conducted in many malaria endemic regions show better sensitivity of RDTs as compared to microscopy [26-29] in malaria diagnosis and it is suggested that RDTs could be used as a supplementary diagnostic tool to aid evidence-based decision making in malaria treatment. The use of RDTs requires neither extensive training [30] nor substantial investment in infrastructure as compared to microscopy. However, there are a number of challenges that need to be addressed for optimal and effective utilization of RDTs in malaria diagnosis in order to provide reliable and credible diagnoses [31]. There should among others be; frequent quality controls and assurances, optimal storage conditions as well as updates on newly available or improved RDTs. If proper instructions are not given to staff especially in the rural communities on how to properly handle these RDTs, their expected usefulness as an alternative diagnostic tool for malaria diagnosis would be highly compromised.

The performance of Parascreen™ has previously been assessed under field conditions [32] involving children with clinical suspicion of malaria in a rural area of Kenya and gave results that were in agreement with other malarial diagnostic tests. Likewise, Paracheck Pf® and ParaHIT®f have also been assessed in community studies within the study area [33,34]. However, during these cross-sectional and longitudinal community studies [33] it has been shown that Paracheck Pf® and ParaHIT®f which are HRP-2 based RDTs were not very sensitive in diagnosing parasite densities of less than 200 asexual stages/μl in asymptomatic children. The sensitivity of different RDTs can also be improved by increasing the concentration of detection antibodies. This is an important component in the manufacturing process, which is usually coupled by frequent evaluations of test performances. Nevertheless their use, despite the low sensitivity at very low parasitaemia has significantly reduced over-prescription of anti-malarials among individuals without malarial infection and they performed well in diagnosing those with symptoms of malaria [5].

Many studies that assess the sensitivity and specificity of RDTs of malaria mainly utilize microscopy as the gold standard [35-37]. However, RDTs detecting histidine rich protein 2 (HRP-2) have the problem of detecting HRP-2 antigen circulating in the blood more than two weeks after IEs have been cleared from the blood stream, resulting in high false positive rates [38,39]. In the study presented here, RDTs that detect both HRP-2 and pLDH as well as HRP-2 only antigens were used in order to detect *P. falciparum* infections, and to also identify non-*falciparum* species in the area [40].

Other sensitive alternative tests superior to microscopy and RDTs such as PCR and real-time quantitative nucleic acid sequence-based amplification (real time QT-NASBA) are also available [28,41], but they are mainly being utilized for epidemiological studies rather than facilitating treatment.

Failure to detect asymptomatic and sub-microscopic infections may leave a large part of the population with untreated infections that may lead to persistent maternal anaemia [42,43] and adverse consequences for the foetus. Simple and easy to use malarial diagnostic tools with adequate sensitivity such as RDTs are therefore required [44] for effective management of PAM. As part of a study entitled strategies to prevent pregnancy-associated malaria (STOPPAM), a sub-study with the aim of assessing the reliability of RDTs in diagnosing PAM was conducted.

## Methods

### Study design

A prospective cohort study on pregnant women was conducted from September 2008 to October 2010. Enrolled women had gestational age of ≤ 24 weeks, were residents within an accessible area of Korogwe District in order to facilitate follow up, had given written informed consent to participate and were willing to deliver at Korogwe District Hospital (KDH). After inclusion, the cohort was followed up through three pre-scheduled antenatal clinic visits (every 2–6 weeks depending on gestational age) until delivery at KDH and satellite outreach dispensaries within Korogwe District, and they were also seen outside the pre-scheduled visits (at any time) whenever necessary. Venous blood samples were collected during each visit and placental blood at delivery for detection of malaria parasites and evaluation of haematological and other biological parameters. Ultrasound sonography was performed at inclusion to ascertain the gestational age and at each antenatal visit to assess intrauterine foetal growth.

### Study site

This study was carried out in Korogwe District, north-eastern Tanzania. The district is inhabited by approximately 261,004 individuals, with a growth rate of 1.4% per annum according to the 2002 Tanzanian human population census report [45]. The district can be topographically stratified into lowland and highland zones, and malaria transmission is perennial with the highest transmission in the lowlands and after long rains [46,47] and low transmission in the highlands at the onset of short rains. *P. falciparum* is the dominant malaria species transmitted by *Anopheles gambiae* s.s and *Anopheles funestus*[48] with entomological inoculation rates of 91 infective bites/person per year [46]. The district has been under constant surveillance of malaria since 2003. Of late, there has been a progressive decline in malaria parasite slide positivity rates in the area [49,50] that transformed the area from hyper-endemic to meso/hypo endemic. Obstetric care is provided at KDH, and at other Health Centres and dispensaries within Korogwe District. More details about the study area can be found in Mmbando *et al.*[49]. According to the 2004–2005 demographic and health survey (DHS) report, the coverage of intermittent preventive treatment during pregnancy with sulphadoxine-pyrimethamine (IPTp-SP) in Tanga Region where Korogwe District is situated was 61.9% [51] as opposed to 90% during the STOPPAM study indicating the importance of sensitization and its impact on utilization of health services.

### Ethical approval, sensitization meetings and informed consent

The study protocol was approved by the Tanzania Medical Research Coordinating Committee with reference number NIMR/HQ/R.8a/Vol. IX/688. Sensitization meetings about the study goals and expectations were held in all catchment villages. All procedures were conducted in consistent with good clinical and laboratory practices. All participants gave written informed consent.

### Study samples

**Figure 1:**
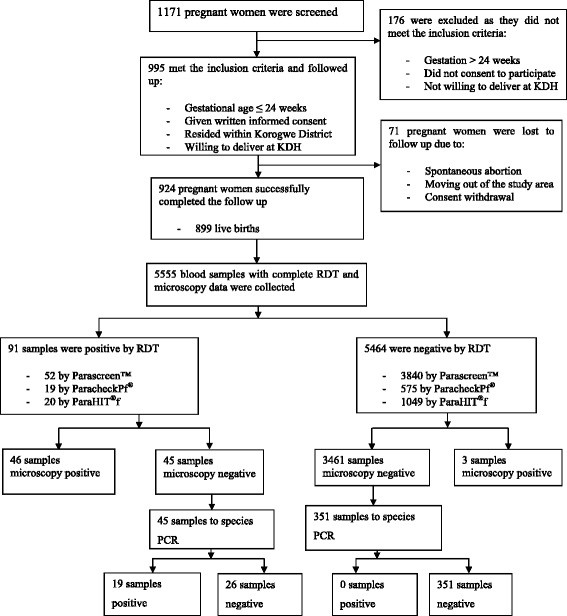
**Consort flow diagram showing sampling strategy for different samples and their subsequent analyses.** RDT: rapid diagnostic test; +ve = positive; -ve: negative; PCR: polymerase chain reaction; KDH: Korogwe District hospital.

A cohort of 924 pregnant women was followed up from enrolment until delivery, and a total of 5,905 samples were collected. Of those, 5,167 venous and 388/650 placental blood samples, for which there were complete RDT and microscopy datasets, were randomly selected for analysis. The calculation of the sample size for the primary study “STOPPAM” was based on the placental hospital study that was previously conducted at KDH and reported a placental parasite prevalence ranging from 10 – 18%. The study screened 1,171 and excluded 176 pregnant women to have the final sample size of 995 pregnant women meeting the inclusion criteria for enrolment. Seventy one pregnant women were lost to follow up due to various reasons (Figure 1).

### Blood drawing

Five to ten ml of venous blood was drawn at inclusion, at scheduled and unscheduled antenatal clinic visits, and just before/after delivery. Ten ml of unperfused placental blood was also collected within 15 minutes of delivery for women who gave birth at the labour ward of KDH. The venous and placental blood for malaria thick and thin blood smear preparations as well as filter paper blood spots for DNA extraction were collected in ethylenediamine-tetraacetic acid (EDTA) minicollect tubes, whereas for RDTs whole blood was directly added to the test device.

### Malaria rapid diagnostic tests

Parascreen™ (Zephyr Biomedicals Goa, India) an RDT that detects histidine rich protein 2 (HRP-2) antigen from *P. falciparum* and parasite lactate dehydrogenase (pLDH) from the *Plasmodium* species was used for the majority of samples in this study. A minority of samples were tested by Paracheck Pf® (Orchid Biomedical Systems –Mumbai, India) or ParaHIT®f (Span diagnostics Ltd – Surat, India), the commonly available RDTs in the study area. Paracheck Pf® and ParaHIT®f only detect HRP-2 antigen from *P. falciparum*. All tests were performed following the manufacturer’s instructions. The laboratory and clinical personnel were trained on how to perform and interpret the RDTs results. As of the 2011 WHO round 3 data on malarial RDTs performances, all the RDTs used in this study have been shown to perform well in all three rounds of testing for performances.

### Microscopic examination of thick and thin blood smears

Thick and thin blood smears from whole EDTA venous and placental blood samples were prepared on glass slide, air dried, stained for 30 minutes with 5% Giemsa stain, then washed gently in tap water, air dried and finally examined with a 100x objective lens under oil immersion. Before a slide was declared negative 100 microscopical thick film fields were scanned. Asexual parasite density was recorded as number of asexual stage parasites per 200 leucocytes, and converted to parasite count per microlitre, by using the actual count of leucocytes as estimated by Sysmex KX-21 N haematological analyser (Kobe, Japan). If the parasite count was less than 10, it was recorded per 500 leucocytes. All slides were read twice by two independent experienced microscopists and results from the two readings with a difference of less than 50% were considered definitive. Smears with discordant results were re-examined by a third experienced microscopist (blinded to the first two readings) and results from two readers that were in agreement were considered final. All laboratory technologists reading the blood slides participated in the proficiency microscopy examination and were certified by the National Institute for Communicable Diseases (NICD), South Africa.

### *Plasmodium* species diagnostic PCR

In order to circumvent the problem of false negatives due to the inability of microscopy in detecting sub-microscopic infections or false positives due to the detection of circulating HRP-2 antigen by RDT even after parasite clearance, all the RDT positive but microscopy negative samples as well as a proportion of both the RDT and microscopy negative samples were analysed using nested *Plasmodium* species diagnostic PCR assay. Fifty microlitre of EDTA blood was added on a premade template of Whatman number 3 filter paper and allowed to dry at room temperature and stored in silica gel to preserve the DNA integrity. Briefly, half sector of the filter spot was excised and incubated with 0.5% saponin (SIGMA™) in 1x PBS and incubated overnight to remove the haemoglobin. The DNA was extracted by Chelex 100 resin method as explained by Wooden *et al.*[52] with some modifications. The DNA supernatant was carefully transferred to a new 96 wells PCR plate without touching the Chelex 100 resin and stored at −20°C until use. The parasite DNA was amplified by outer and nested species diagnostic PCR according to Snounou *et al.*[53] and the PCR products were analysed in 1.5% ethidium bromide stained UltraPure™ agarose gel (Invitrogen) with a Gene ruler™ 50 bp DNA ladder (Lonza, Belgium). The gels were visualized under UV trans-illuminator from BIO-RAD.

### Management of malaria

All women with confirmed malarial infection based on RDTs and/or microscopy were treated with anti-malarial drugs. Uncomplicated and asymptomatic infections were treated by administration of quinine in the first trimester and artemether-lumefantrine during the second or third trimester.

### Data management and analyses

All data were documented on case record forms and double entered into Microsoft Access database, cleaned, validated and transferred into R. version 2.12.0 statistical package for analyses. Statistical significance level was considered at α = 0.05. Baseline characteristics (demographic, clinical and parasitological) were analysed using descriptive statistics. Sensitivity was defined as the proportion of true malarial cases (positive blood smears and/or PCRs) that were correctly identified by positive RDTs whereas specificity was the proportion of true negative malarial cases (negative blood smears/negative PCRs) that were correctly identified by negative RDTs. Positive predictive value was the proportion of true malarial cases (positive blood smears and/or PCR) among the individuals with the positive RDTs. Negative predictive value was the proportion of true negative malarial cases (negative blood smears/PCRs) among the total number of negative RDT tests. Accuracy was defined as the proportion of all tests that gave correct results (True Positive + True Negative)/ number of all tests.

## Results

In total, 1,171 pregnant women were screened, 995 (85%) met the inclusion criteria and were enrolled and followed up. Seventy-one women were lost to follow up or excluded before delivery due to spontaneous abortion or moving out of the study area. Of the enrolled cohort, 924/995 (93%) successfully completed follow up, from whom 5,555 blood samples with complete RDT and microscopy data were selected for further analyses (Figure 1). Of the enrolled cohort, 471 women were primi/secundigravid and 524 were multigravid with mean ages of 22.6 ± 4.2 and 30.7 ± 5.3 years for primi/secundigravid and multigravid women, respectively. There were 899 successful live births among the 924 pregnant women who successfully completed the follow up. *P. falciparum* was the only malarial parasite detected by microscopy. In women with a positive slide reading, the median asexual parasite density/μl was 2,090 [range; 40–390,748] and 4,163 [range; 40–45, 760] for primi-/secundigravid and multigravid, respectively, (Table 1). 3,892 samples were tested with Parascreen™, an RDT which detects both *P. falciparum* and non-*falciparum* parasites. Thirteen samples (0.3% [95% CI: 0.2 – 0.6] were RDT positive for non-*falciparum* species, and of these 11 were also RDT-positive for *P. falciparum*. As stated above non-*falciparum* parasites were not detected by slide reading and the remaining part of the article only compares detection of *P. falciparum* by the different methods employed since it is the one mainly involved in PAM pathogenesis.

**Table 1:** Age, mean haemoglobin and parasite density of positive slides among the pregnant women included in the study

**Parameter**	**Gravidity**	
	**Primi/Secundigravid (n = 471)**	**Multigravid (n = 524)**
Age in years (Mean ± SD)	22.6 ± 4.2	30.7 ± 5.3
Mean haemoglobin level (g/dL) [range]	10.45 [3.1 – 22.8]	10.53 [3.8 – 22.4]
Median asexual parasite density/μl [range]	2090 [40–390748]	4163 [40–45760]

SD: standard deviation; dL: deciliter; μl: microlitre.

### Comparison of rapid diagnostic test with microscopy results

Overall 91/5,555 (1.6% [1.3-2.0]) samples were positive for malarial parasite antigen based on RDTs whereas 49/5,555 (0.9% [95% CI; 0.7 –1.1]) were positive by microscopy (Table 2). Of the 91 RDT positive samples, 46 (50.5% [95% CI: 40.5 - 60.6] were microscopy positive whilst 45 (49.5% [95% CI; 39.4 – 59.5]) were microscopy negative. To test whether the 45 microscopy negative but RDT positive samples were genuinely negative or false positive, a species diagnostic PCR assay was performed. Three (0.05% [0.02 - 0.2]) samples were positive by microscopy but negative by RDT. These samples were not tested by PCR assay, but the slide readings were confirmed by two independent expert slide readers as positive with median parasite density of 224.75 [range; 86 – 486].

**Table 2:** Performance of different brands of rapid diagnostic tests in diagnosing PAM

**RDT type**	**No. of Samples**	**Results**				**Parasite density (asexual stages/μl)**	
		**Rapid Diagnostic Test**		**Microscopy**		**Median**	**Range**
		**Positive**	**Negative**	**Positive**	**Negative**		
Parascreen™	3892	52 (1.3%)	3840 (98.7%)	28 (0.7%)	3864 (99.3%)	2565	39.5–101208
Paracheck®Pf	594	19 (3.2%)	575 (96.8%)	8 (1.3%)	586 (98.7%)	2004.5	581.5-23587.5
ParaHIT®f	1069	20 (1.9%)	1049 (98.1%)	13 (1.8%)	1056 (98.8%)	1023.63	242.5–390748
**Total**	**5555**	**91 (1.6%)**	**5464 (98.4%)**	**49 (0.9%)**	**5506 (99.1%)**	**2090**	**40–390748**

RDT: rapid diagnostic test; μl: microlitre.

### PCR confirmation of the RDT positive but microscopy negative cases

The 45 RDT positive but microscopy negative samples were checked by nested PCR to ascertain whether they were sub-microscopic infections. Interestingly, 19/45 (42.2% [95% CI; 29.0 - 56.7] were positive by PCR (Table 3).

**Table 3:** PCR analysis results of samples that were RDT positive but microscopy negative

**RDT type**	**No. microscopy negative samples**	**PCR results**	
		**Positive**	**Negative**
Parascreen™	30	12 (40.0%)	18 (60.0%)
ParacheckPf®	9	6 (66.7%)	3 (33.3%)
ParaHIT®f	6	1 (16.7%)	5 (83.3%)
**Total**	**45**	**19 (42.2%)**	**26 (57.8%)**

PCR: polymerase chain reaction; RDT: rapid diagnostic test.

### Nested species diagnostic PCR correction of RDT and microscopy negative samples

A proportion 351/650 (54%) of the available placental blood samples that were malarial negative by both RDT and microscopy were checked by nested PCR targeting *P. falciparum* as it is the most prevalent species in the study area and the one responsible for PAM pathology. Only 650 placental blood samples could be collected due to clotted placental blood (e.g. retained placenta or the need for the project nurses to stay in the labour room to resuscitate the newborn, delaying the transportation of the placenta to the laboratory for processing) and 190 women gave birth outside the KDH setting making it impossible to collect placental blood. Due to limited resources and time, only a small proportion of all the negative samples could be checked by PCR. The 351 placental instead of venous blood samples were randomly selected as microscopy could have missed some parasites in the peripheral blood due to parasite sequestration in the placenta and also due to the presence of debris and other contaminants in the placental blood that might have resulted to poor quality blood smears not easily readable. Interestingly, all these samples were negative by PCR indicating that both microscopy and RDTs performed equally well in diagnosing true negative cases.

### Performance of different RDTs after PCR correction

The performance of the different RDTs and microscopy was compared in the 442 samples in which PCR was performed and using the PCR results as the golden standard (Table 4). There was no statistically significant difference between the performances of the different RDTs. However, the study was not designed to directly compare the different RDTs and these RDTs were not employed on the same samples. All the PCR and slide positive samples were *P. falciparum.* Probably the use of a modified Snounou PCR using *P. ovale. wallikeri* and *P. ovale. curtisi* primers might have increased the number of positive PCRs and hence the overall performance of the RDTs.

**Table 4:** Comparison of RDT and microscopy performance using PCR as golden standard in the 442 samples where PCR results were available

**Diagnostic category**	**No. of samples**	**Sensitivity (%)**	**Specificity (%)**	**PPV (%)**	**NPV (%)**
		**% [95% CI]**	**% [95% CI]**	**% [95% CI]**	**% [95% CI]**
Parascreen™	301	100.[89.9–100.0]	93.3 [89.6 -95.7]	65.4 [51.8-76.9]	100[98.5- 100.0]
Paracheck Pf®	64	100 [8 0.6-100]	93.8 [83.2 – 97.9]	84.2 [62.4-94.5]	100 [92.1-100.0]
ParaHIT®f	77	100 [79.6–100.0]	91.9 [82.5– 96.5]	75.0 [53.1 – 88.8]	91.9 [84.9-95.8]
Microscopy	442	70.8 [58.0 –81.1]	93.1[89.9-95.4]	63.9 [51.2-74.6]	94.5 [92.0–96.8]

PPV: positive predictive value; NPV: negative predictive value; 95% CI: 95% confidence interval.

## Discussion

The performance of RDTs in diagnosing PAM was evaluated against microscopy and nested *Plasmodium* species diagnostic PCR in a cohort of pregnant women in north-eastern Tanzania. The use of RDTs might act as an appropriate complementary diagnostic tool for malaria instead of only relying on presumptive treatment based on clinical grounds in areas with limited expert microscopy and laboratory infrastructure. Prescription of any drug during pregnancy is a challenging task due to potential risks of harming the foetus [54], over-prescription and subsequent risk of drug resistance development [55]. Simple, cheap, reliable, accurate, easy to use, sensitive and specific diagnostic tests that can identify genuine malarial cases are the only means of allowing accurate malaria detection and rational treatment. With the escalating anti-malarial drug resistance which necessitates the deployment of expensive artemisinin-based combination therapy, there is a need to prescribe anti-malarial drugs only to patients with true malarial illness [56]. The present study demonstrates that RDTs can act as a diagnostic tool to manage malaria during pregnancy in resource poor settings with limited access to expert microscopy as they are easy to use and perform better than microscopy. Based on the PCR results, the different types of RDTs used in this study were able to capture over 40% of sub-microscopic infections missed by microscopy.

According to the current Ministry of Health and Social Welfare’s policy in Tanzania on malaria diagnosis, the use of RDTs is not yet implemented as a routine practice for pregnant women at antenatal clinics. Therefore, there is a need to provide evidence-based data on the best diagnostic supplement/alternative for malaria diagnosis during pregnancy in areas with limited laboratory infrastructure. This will assist the National Malaria Control Programme in Tanzania and beyond when considering RDTs as a possible routine diagnostic tool in malaria diagnosis during pregnancy. According to the recent WHO report [57] on the performance of different RDTs, it has been shown that they are easy to use, are heat stable and have the ability to detect parasitaemia as low as 200 asexual stages/μl. This makes RDTs an ideal diagnostic supplement to malaria diagnosis in resource constrained settings.

In many of the malaria endemic regions including Tanzania, healthcare delivery in peripheral settings is compromised by the lack of well equipped laboratories and personnel with sufficient expertise in malaria microscopy [58]. Microscopy could be as sensitive as RDTs or even more sensitive when done well. However, adequate infrastructure, maintenance of good quality microscopy and proper training on expert microscopy are not always present in many malaria endemic settings. Mismanagement of sub-microscopic infections could result in low but persistent parasitaemia that may culminate in adverse pregnancy outcomes [42,43]. Under-diagnosis and/or wrong diagnosis of true malarial infections may lead to infections going untreated or being wrongly treated as non-malarial illnesses, with subsequent adverse pregnancy outcomes and/or acting as potential reservoirs of transmission. However, in all malaria endemic settings, children with febrile illnesses are treated by anti-malarials and/or other antimicrobials following the World Health Organization guidelines’ on integrated management of childhood illnesses.

Singer *et al.*[59] have shown that microscopy underestimates the real malarial burden during pregnancy. Nonetheless in their study, contrary to this study, PCR detected more positive cases as compared to RDTs, whilst assessing only placental blood samples. The present study might differ from that of Singer and colleagues in malaria transmission intensities and also in the current study only a small proportion of RDT and microscopy negative samples were checked by PCR due to limited resources and time. However, when taken as a proportion of placental blood the 351 samples checked by PCR accounts for 54% of the available placental blood samples with complete data. The message portrayed here is that microscopy was shown to have underestimated the true malarial prevalence after PCR correction.

The persistence of HRP-2 circulation in the blood more than two weeks even after successful clearance of IEs in the bloodstream is one of the concerns on the usefulness of HRP-2 based RDTs in malaria diagnosis, as has been reported by many studies [35,36,38,39,60]. However, in most of these studies microscopy was used as gold standard without PCR correction and this might have categorized sub-microscopic infections as false positives due to the limited sensitivity of microscopy. In the present study, all the RDT positive but microscopy negative samples were checked by PCR and the analyses showed that a large proportion of the RDT positive but microscopy negative samples were in fact sub-microscopic infections. Treatment of these few false positive women with anti-malarials might provide some prophylactic effect against subsequent infections outweighing the risk of not treating genuine sub-microscopic infections missed by microscopy that could have a profound effect on the pregnancy outcome. The relatively poor performance of microscopy compared to RDT cannot be explained by suboptimal conditions for microscopy as the present study was conducted in parallel with a large clinical trial [61]. Therefore, laboratory conditions were excellent and all the laboratory technologists had ample experience in malaria diagnosis and were undergoing proficiency microscopy tests on a regular basis.

The performance of RDTs in malaria diagnosis in the present study is in agreement with studies by Tjitra, Batwala and Tham *et al.*[26,27,62] showing that RDTs were performing better than microscopy in malaria diagnosis under field conditions. Likewise, Bell *et al.*[38] conducted a study in the Philippines in an area of low endemicity and reported that sub-microscopic infections missed by microscopy but captured by RDTs were actually true infections after PCR correction. On the other hand, the current study is not in agreement with a study by Schachterle *et al.*[63] that showed that RDTs had high rates of false positives and negatives in a region of hypoendemicity. However, the results of that study were purely based on microscopy data without PCR correction.

RDTs missed some few infections, which were positive in repeated microscopic investigations. This could be due to assay degradation as a result of humid conditions or batch variability of the RDTs [64], delay in HRP-2 surge after increased parasite density [38] or due to deletion of *hrp-2* genes in some parasites [60]. Some studies have also reported reduced sensitivity of RDTs as a result of low parasitaemia [33,65] and this could explain the reason for the few cases with low parasite densities missed by RDTs [49,50].

This study indicates that RDTs outperform expert microscopy in detecting asymptomatic *P. falciparum* in pregnant women. Given the difficulties in establishing reliable microscopy based diagnostic services, RDTs are good alternative for the detection and in the management of *P. falciparum* infections in pregnant women. RDTs can both be used to detect infections not cleared by IPTp or to detect infections where the malaria endemicity is too low to warrant IPTp.

## Competing interests

All authors declare no any conflict of interest.

## Authors’ contributions

DTRM, CS, JL, TT, AS, ML, PD, AJFL and MA designed the study. DTRM, JL, CS, MO, SB, CP, DJ, PM, DA and MA conducted the study and participated in the laboratory analyses. DTRM drafted the manuscript. All authors reviewed the manuscript and provided critical inputs. All authors read and approved the final manuscript.
